# Anticytotoxic T-Lymphocyte Antigen-4 Induced Autoimmune Hypophysitis: A Case Report and Literature Review

**DOI:** 10.1155/2015/570293

**Published:** 2015-01-28

**Authors:** Deborah Majchel, Mary T. Korytkowski

**Affiliations:** Division of Endocrinology and Metabolism, Department of Medicine, University of Pittsburgh Medical Center, 3601 Fifth Avenue, Suite 3B, Pittsburgh, PA 15213, USA

## Abstract

*Objective*. We describe a case of autoimmune hypophysitis induced by the anticytotoxic T-lymphocyte antigen-4 (CTLA-4) agent, ipilimumab. *Methods*. Case presentation and review of the literature. *Results*. Autoimmune hypophysitis, a previously described rare disorder, is being recognized more frequently as a side effect of novel immunomodulatory agents used in the treatment of malignancies such as melanoma. CTLA-4 agents are associated with immune-related adverse effects (irAE) which occur as a result of activation (or lack of inactivation) of the immune response. This impacts not only malignant cells but also different host organ-systems. Autoimmune hypophysitis is one of several endocrinopathies associated with these agents. *Conclusion*. It is important that endocrinologists become familiar with the endocrinopathies, such as autoimmune hypophysitis, associated with new immunomodulator agents which are being used with increasing frequency to treat a variety of malignancies.

## 1. Introduction

New human monoclonal antibodies that target the T cell receptor, cytotoxic T-lymphocyte antigen-4 (CTLA-4), are associated with immune mediated adverse events (irAE). Classic lymphocytic hypophysitis is an uncommon disorder which is autoimmune in nature. This novel class of immunomodulators being used for the treatment of certain malignancies, such as melanoma, has led to an increase in the diagnosis of hypophysitis, which is part of its side effect profile.

We describe a case of ipilimumab-induced hypophysitis.

## 2. Case Presentation

A 31 year-old female who had undergone excision of a stage IIIB melanoma of the right heel with inguinal node dissection at an outside hospital presented to the emergency department with a 9-day history of intermittent temporal headaches. Postoperatively, she had been enrolled in an open label research protocol for which she was randomized to high dose ipilimumab (10 mg/kg). At time of presentation she had received 3 doses of this agent at 3 week intervals. Her most recent dose was administered 2 weeks prior to her ER presentation. Her only side effect with the first two doses was pruritus.

In the emergency department, she described her headaches as nonradiating and initially relieved by nonsteroidal agents. Two days prior to presentation, the headaches became more severe (described as 8/10 in intensity), constant, and unrelieved by nonsteroidal agents. Her review of systems was positive for weight gain of 15 pounds over nine weeks and previous history of sore throat and palpitations. She denied changes in vision, galactorrhoea, temperature intolerance, anxiety, or depression. Her only other medication was a Mirena intrauterine device (IUD). On physical exam, she was afebrile with a normal blood pressure (126/83 mmHg), pulse of 92 beats/minute, and respiratory rate of 16 breaths/minute. She was alert and in no distress. Visual field testing by confrontation was normal. Aside from right inguinal and right heel scars from her recent surgery, her exam was normal.

**Figure 1:**
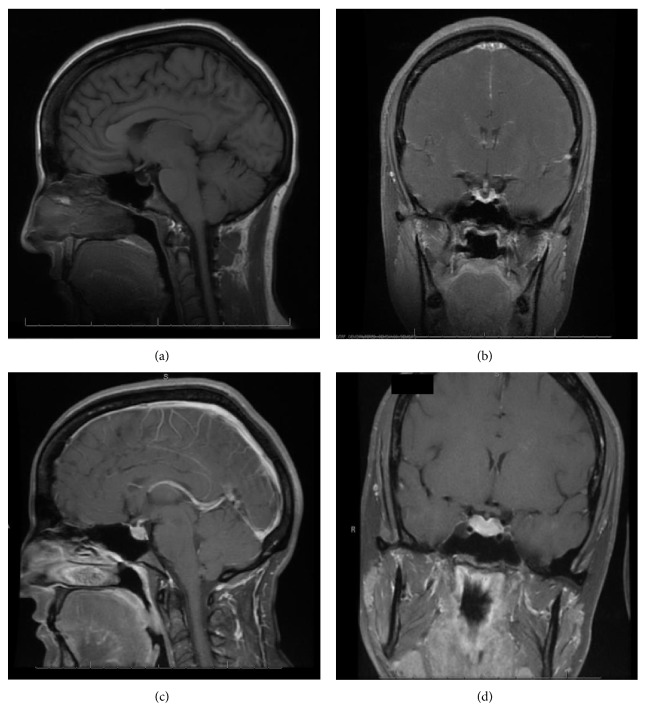
Case MR images. Sagittal (a) and coronal (b) unenhanced images of the pituitary, revealing a slightly atrophic pituitary gland. Two months later, at presentation, sagittal (c) and coronal (d) postcontrast images reveal an enlarged pituitary size with diffuse enhancement of the pituitary and infundibulum without focal lesion.

With the exception of a mild leukocytosis (12.9 K), initial laboratory findings demonstrated normal chemistry and hematology panels. Hormonal studies were pending, although she was noted to have a suppressed TSH and elevated free T4 2 weeks prior to admission (Table 1). Given the severity of her symptoms as well as awareness of possible hypophyseal involvement with anti-CTLA-4 therapy, the decision was made to obtain an MRI of the pituitary. This demonstrated a gland size at the upper limit of normal, with a measured increase in size from 3 × 15 mm to 9 × 21 mm when compared to a study done 2 months earlier as part of restaging workup (Figure 1). Based on these findings, a presumptive diagnosis of hypophysitis was made and therapy with high dose intravenous methylprednisolone was initiated.

## 3. Discussion

**Figure 2:**
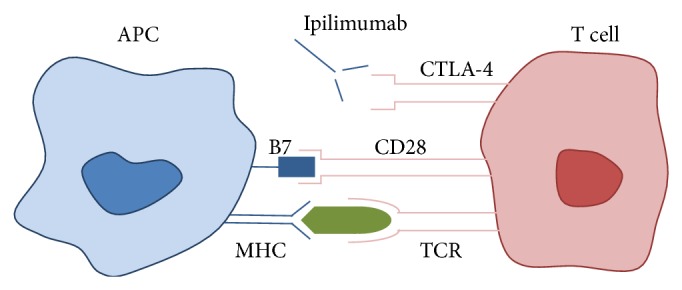
APC: antigen presenting cell. MHC: major compatibility complex. TCR: T cell receptor. Adapted from Kähler and Hauschild [1].

Ipilimumab and tremelimumab are human monoclonal antibodies that act by inhibiting the binding of B7 to CTLA-4, thus preventing inactivation of the immune response. T cell activation begins when the T cell receptor (TCR) binds to an antigen presented by the antigen presenting cell (APC) via the major histocompatibility complex (MHC) (Figure 2). B7, a ligand found on the APC, binds to its receptor on the T cell (CD28), which is the second signal needed for T cell activation and induction of the immune response. After a period of 48–72 hours, the CTLA-4 receptor is upregulated and migrates to the T cell surface. B7 binds preferentially to the CTLA-4 receptor, leading to T cell inactivation and downregulation of the immune response. In the presence of CTLA-4 inhibitors, the net effect is continued proliferation of activated T cells, increased antitumor activity, and possible irAE.

Persistent T cell activation and propagation of the immune response leads to targeting not only tumor cells but also normal host organ-systems, a term referred to as irAE. These occur in a sequential manner, with the most commonly affected systems being the skin at 3-4 weeks, gastrointestinal (GI) tract and liver at 6-7 weeks, and endocrine system at 9 weeks [1]. The described endocrinopathies include hypophysitis with an incidence of ~5%, thyroid dysfunction (hypothyroidism or hyperthyroidism) with an incidence of 0–4%, and primary adrenal insufficiency with an incidence of 0.3–1.5% [3]. These toxicities can be graded according to severity on a scale from 1–5 (Table 2). Most irAE occur during the 12-week induction phase but can also happen weeks to even months after stopping therapy [4]. Pooled analysis from phase I–III trials demonstrates that up to 72% of patients develop an irAE at the higher dosing range which the patient in this case was receiving [1, 3, 5] (Table 3).

Patients who develop anti-CTLA-4 associated endocrinopathies may present with nonspecific symptoms such as fatigue, weakness, headache, nausea, behavioral changes, visual impairments, memory loss, decreased libido, anorexia, insomnia, and cold or heat intolerance. A high index of suspicion is required to prompt endocrine evaluation and therapeutic intervention in patients who present with any of these symptoms, with particular urgency in the setting of pituitary or adrenal insufficiency [1, 3, 5–7].

Evaluation should include assessment of pituitary function with morning (8 am) cortisol (if possible), adrenocorticotropic hormone (ACTH), and cosyntropin stimulation testing. Testing of the hypothalamic-pituitary-thyroidal axis includes measurement of free T4, thyroid stimulating hormone (TSH), with a free T3 in some situations. Testing of the gonadal axis is indicated in the presence of hypogonadal symptoms or amenorrhea with measures of follicle stimulating hormone (FSH), luteinizing hormone (LH), prolactin, testosterone (in men), and estradiol in women. The described sequence of pituitary dysfunction is impaired secretion of ACTH, then TSH, followed by loss of FSH, LH, and growth hormone (GH). Prolactin can be low or high. Diabetes insipidus rarely develops and, when present, raises suspicion for infiltration of the pituitary stalk by tumor [3, 6].

The patient in this case had hormonal evidence of both thyroiditis and hypophysitis, despite the absence of symptoms of hyperthyroidism or hypopituitarism. The presentation of a severe headache prompted evaluation of pituitary hormonal function and imaging. Hormonal testing revealed a detectable but low cortisol level (drawn at 4 pm) with a normal ACTH. It would be expected that a patient presenting with a headache severe enough to prompt an ER visit would have a higher cortisol. Unfortunately, treatment with steroids was started prior to obtaining a cosyntropin stimulation test. Her repeat TSH remained low with a now normal free T4, suggesting the possibility of resolving thyroiditis. Her report of occasional palpitations at the time of the elevated free T4 lends support to this hypothesis.

MRI findings of suspected hypophysitis are often nonspecific. These include diffuse enlargement of the pituitary, which can be missed unless earlier imaging studies are available for comparison. Other imaging characteristics can include a homogeneous or heterogeneous enhancement of the gland with or without an extension of the inflammatory process towards the stalk [1, 7, 8]. It is important to note that a normal appearing gland on MRI does not exclude hypophysitis [7].

As the number and types of cancers treated with these newer immunomodulatory agents increase, there is an associated rise in reported cases of hypophysitis (Table 4). Given the frequency and consistency with which these autoimmune adverse effects appear, many physicians advocate screening all patients treated with anti-CTLA-4 agents for endocrinopathies with measurements of morning serum cortisol level, electrolytes (to detect hyponatremia or hyperkalemia as indicators of adrenalitis), TSH, and free T4 levels. One study described the incidental finding of hypophysitis on an ^18^F-FDG PET-CT done as part of the evaluation of metastatic lesions in a 77-year-old man with nodular malignant melanoma, prior to any clinical or laboratory abnormalities. However, the use of this imaging technique is not validated for the purpose of screening [9].

Classic lymphocytic hypophysitis histologically appears to be autoimmune in nature. This has not been consistently demonstrated with anti-CTLA-4 hypophysitis as many if not most of these patients will not have a biopsy performed. However, most of the clinical and radiological features of ipilimumab associated hypophysitis are consistent with the classical form, including its response to glucocorticoids, which is the standard treatment of both [10].

Once anti-CTLA4 hypophysitis is diagnosed, treatment with steroids is recommended. Suggested regimens include methylprednisolone or prednisone at 1-2 mg/kg/day or dexamethasone 4 mg q6h for 1 week, tapered over a course of 4 weeks. High dose glucocorticoid treatment does not appear to decrease the antitumor effect of CTLA blockade; in fact, a trend between the development of immune-related adverse effects and successful treatment with CTLA-4 antibodies has been suggested [1, 3, 5].

Patients may also require thyroid or gonadal hormone replacement with close followup to determine if there has been resolution of any hormonal deficiencies. The time that is required for symptoms to resolve and the need for continuous hydrocortisone replacement therapy is approximately 20 weeks but can be longer and in some cases it is even lifelong [5].

Treatment with ipilimumab should be held for grades 1-2 hypophysitis and then can be safely resumed at a later date. It is recommended that treatment with these agents be discontinued for grades 3-4 irAE. In the case presented, a repeat MRI done one week after initiating steroid treatment in our patient demonstrated a reduction in the size of her pituitary, allowing ipilimumab to be resumed.

## 4. Conclusion

Lymphocytic hypophysitis was previously considered a rare cause of pituitary dysfunction. However, the incidence of this disorder has increased with the introduction of immunomodulating chemotherapeutic agents. The anti-CTLA4 class of immune modulators (ipilimumab) has a mechanism of action that predisposes patients to immune-related endocrinopathies. Autoimmune hypophysitis is one of several endocrinopathies described with use of these agents. It is important that endocrinologists become familiar with this possible range of side effects as a way of allowing early diagnosis and prompt initiation of therapy, which in some cases could be lifesaving.

## Figures and Tables

**Table 1:** Laboratory findings.

	Reference values	16 days PTA	Admission
ACTH	9–46 pg/mL		38
Cortisol (random)	N/A (*μ*g/dL)		7
TSH	0.3–5.0 *μ*IU/mL	0.053	0.046
Free thyroxine (T4)	0.8–1.8 ng/dL	2.31	1.26
FSH	0.3–10.5 mIU/mL		1.5
LH	N/A (mIU/mL)		0.8
Prolactin	0.6–20 ng/mL		18.7

ACTH: adrenocorticotropic hormone.

TSH: thyroid stimulating hormone.

FSH: follicle stimulating hormone.

LH: luteinizing hormone.

PTA: prior to admission.

**Table 2:** Toxicity grading and endocrine adverse events associated with immune checkpoint inhibitors, according to Common Terminology Criteria for Adverse Events (CTCAE) of National Institutes of Health (National Cancer Institute) [2].

Endocrine adverse event	Grade	Description
Hypothyroidism	1	Asymptomatic; clinical or diagnostic observations only; intervention not indicated
	2	Symptomatic; thyroid replacement indicated; limiting instrumental activity of daily living (ADL)
	3	Severe symptoms; limiting self-care ADL; hospitalization indicated
	4	Life-threatening consequences; urgent intervention indicated
	5	Death

Hyperthyroidism	1	Asymptomatic; clinical or diagnostic observations only; intervention not indicated
	2	Symptomatic; thyroid suppression therapy indicated; limiting instrumental activity of daily living (ADL)
	3	Severe symptoms; limiting self-care ADL; hospitalization indicated
	4	Life-threatening consequences; urgent intervention indicated
	5	Death

Adrenal insufficiency	1	Asymptomatic; clinical or diagnostic observations only; intervention not indicated
	2	Moderate symptoms; medical intervention indicated
	3	Severe symptoms; hospitalization indicated
	4	Life-threatening consequences; urgent intervention indicated
	5	Death

Hypophysitis	1	Asymptomatic or mild symptoms; clinical or diagnostic observations only; intervention not indicated
	2	Moderate; minimal, local or noninvasive; intervention indicated; limiting age-appropriate instrumental ADL
	3	Severe or medically significant but not immediately life-threatening; symptoms; hospitalization or prolongation of existing hospitalization indicated; disabling; limiting self-care ADL
	4	Life-threatening consequences; urgent intervention indicated
	5	Death

**Table 3:** Frequency of adverse events with 10 mg/kg dose of ipilimumab.

Adverse event	Any grade (%)	Severe (grades 3-4) (%)
Skin	47–68	0–4
Gastrointestinal	31–46	8–23
Hepatitis	3–9	3–7
Hypophysitis	4–6	1–5

**Table 4:** Cases of anti-CTLA4 induced hypophysitis reported in the literature.

Authors, year	Type of study	*N*	Comments
Min et al., 2014 [11]	Retrospective cohort	25	Evaluated time to onset, frequency of resolution, and the effect of high-dose corticosteroids on clinical outcome

Albarel et al., 2015 [12]	Retrospective (observational)	15	Characterized hypophysitis in terms of clinical signs, hormonal profile, and imaging at time of diagnosis and during long-term follow-up

Chodakiewitz et al., 2014 [13]	Case series	3	Descriptive

Nallapaneni et al., 2014 [14]	Case report	1	Describes a patient who developed uveitis and hypophysitis with anterior and posterior pituitary involvement without MRI findings

Faje et al., 2014 [15]	Retrospective review	17	Descriptive

Ryder et al., 2014 [16]	Retrospective	19	Descriptive

Marlier et al., 2014 [17]	Case series	4	Descriptive

Anderson and Bhatia, 2013 [18]	Case report	1	Descriptive

Lammert et al., 2013 [7]	Case series	7	Discusses screening and management of hypophysitis in patients with metastatic cancer

Corsello et al., 2013 [3]	Literature review	N/A	Review of existing literature on endocrine side effects induced by immune checkpoint inhibitors

Van der Hiel et al., 2013 [9]	Case report	1	Descriptive

Lotem et al., 2012 [19]	Descriptive	N/A	Description of CTLA-4 blockade as immunotherapy for malignant melanoma

Andrews and Holden, 2012 [4]	Descriptive	N/A	Describes characteristics and management of immune related adverse effects related to ipilimumab

Thomsen 2012 [20]	Case series	2	Descriptive

Weber et al., 2012 [5]	Descriptive	N/A	Describes management of immune-related adverse events and kinetics of response with ipilimumab

Juszczak et al., 2012 [21]	Case report and review	1	Descriptive

Torino et al., 2012 [22]	Descriptive	N/A	Describes CTLA-4 induced hypophysitis as a new cause of a previously rare disease

Bronstein et al., 2011 [23]	Case series	2	Describes radiologic manifestations of immune-related adverse events in patients with metastatic melanoma receiving anti-CTLA-4 antibody therapy

Barnard et al., 2012 [10]	Case report	1	Hypophysitis presenting with hyponatremia

Kähler and Hauschild, 2011 [1]	Descriptive	N/A	Reviews mechanisms of action with update on clinical trials and recommendations for managing side effects of anti-CTLA-4 antibody therapy

Boasberg et al., 2010 [24]	Descriptive	N/A	Describes mechanism of action, immune response criteria, and side effect profile of anti-CTLA-4 agents

Dillard et al., 2010 [6]	Case series	2	Patients with prostate cancer who develop hypopituitarism during treatment with ipilimumab

Kaehler et al., 2009 [25]	Case report	1	Descriptive

Carpenter et al., 2009 [8]	Case series	3	MRI findings in 3 patients with ipilimumab induced hypophysitis

Yang et al., 2007 [26]	Case series	2	2 patients with metastatic renal cell cancer and ipilimumab associated hypophysitis
